# Characteristics Associated with Young Adults’ Intentions to Engage with Anti-Vaping Instagram Posts

**DOI:** 10.3390/ijerph20116054

**Published:** 2023-06-05

**Authors:** Jessica Liu, Donghee N. Lee, Elise M. Stevens

**Affiliations:** 1 Department of Social and Behavioral Sciences, Harvard T.H. Chan School of Public Health, Boston, MA 02115, USA; 2 Department of Population and Quantitative Health Sciences, Division of Preventive and Behavioral Medicine, University of Massachusetts Chan Medical School, Worcester, MA 01655, USA

**Keywords:** e-cigarettes, advertising, social media, Instagram, engagement

## Abstract

The purpose of this study was to identify behavioral and sociodemographic factors associated with intentions to engage with anti-vaping Instagram posts among a young adult population. This study proposes the following research questions: (1) Does e-cigarette use status influence intentions to engage with anti-vaping Instagram posts?, and (2) How are e-cigarette use and social media use associated? We recruited a convenience sample of young adults (N = 459; aged 18–30 years) in July of 2022 into an online experimental study from Prolific. Participants saw five image-based Instagram posts about the health harms of using e-cigarettes. Participants were then asked about their intentions to engage (“Comment on”, “Reshare”, “DM/Send this to a friend”, “Like”, and/or “Take a screenshot of”) with the posts. We used logistic regression to run adjusted models for each engagement outcome, which included fixed effects for sociodemographics, tobacco use, and social media/internet use. For the sum of the engagement outcome, we used Poisson regression. Total number of social media sites used was associated with intentions to “Like” the posts (*p* = 0.025) and the overall engagement score (*p* = 0.019), respectively. Daily internet use was associated with intentions to “Comment on” (*p* = 0.016) and “Like” (*p* = 0.019) the posts. Young adults who reported past 30-day e-cigarette use had higher odds of using Twitter (*p* = 0.013) and TikTok (*p* < 0.001), and a higher total number of social media sites used (*p* = 0.046), compared to young adults who reported never use e-cigarettes. The initial evidence from our exploratory research using a convenience sample suggests that social media campaigns about the harms of e-cigarette use may be an effective way to engage younger audiences, a generation that frequents social media. Efforts to disseminate social media campaigns should consider launching on multiple platforms, such as Twitter and TikTok, and consider e-cigarette use status when posting.

## 1. Introduction

Social media are key drivers of recent high levels of e-cigarette use (or vaping), and continue to play a role in proliferating messaging around e-cigarette use to young people [1]. Young people are exposed to official e-cigarette industry content online, as well as social media posts from friends or influencers that encourage substance use, including tobacco use [2,3]. In recent years, discussion and communication around e-cigarettes on certain social media sites, such as Twitter, have greatly increased [4]. Social media use has been associated with e-cigarette use among young people [5,6,7]. However, there lacks research on how young people interact with online and/or social media content, and who is more likely to engage with certain types of online content. Most studies about the use of social media and risk behaviors have focused on high-school-aged youth and alcohol use [8,9], and little have focused on e-cigarettes. Because young adulthood is a time of high exposure to social media and when tobacco use dependency develops [10,11], young adults (aged 18–30) are vulnerable to the health harms and addictive effects of e-cigarettes. Thus, it is important to understand how social media influences young adult perceptions of e-cigarettes.

### 1.1. Literature Review

While social media have contributed to the popularity of e-cigarettes among young people [2,12,13], social media may also be a way to communicate about the risks of e-cigarette use to this age group. Unlike traditional media (e.g., TV and radio), social media allow people to directly engage with messages, such as actively interacting with or responding to social media posts [14,15,16]. Specifically, engaging with e-cigarette content on social media can promote discussions about the health effects of using e-cigarettes among young people; thus, engagement with social media is an important activity to measure. From the limited research base examining engagement and e-cigarette social media messaging, research has found that young people shared memes when they were funny [2], and individuals engaged more with posts comparing e-cigarettes with cigarettes [17]. Other research examining e-cigarette industry sponsored posts and social media messaging have predominately focused on analyzing the content of posts [18,19] or characteristics of posts (e.g., likes, shares) [20].

Given the ubiquity of social media and online messaging, prior research has shown the need to promote health-conscious attitudes about various health behaviors on social media, especially among young adults [21]. One important area is in the context of e-cigarette use, given the rise of health misinformation in recent years [22,23,24] and the popularity of new and emerging tobacco products [17,25,26]. For instance, social media may be able to serve as a conduit to promote discontinued use of e-cigarettes or serve to message about prevention in general. Prior qualitative studies involving young adults have reported that online content and social media present substances, especially e-cigarettes, in positive and humorous ways [2], which can decrease risk perceptions of these products. Additionally, research has found associations between viewing online content promoting the use of substances, such as tobacco, and subsequent use of that product, especially among young adults [8,14]. In other words, it is important to understand what content on social media can dissuade young adults from using e-cigarettes. Thus, more research is needed to understand the association of social media use and young adults’ perceptions of e-cigarette social media content.

The U.S. Food and Drug Administration and other public health organizations have made efforts to communicate the health harms of e-cigarettes [27]. Yet, evidence has also shown that young adults who use tobacco products may avoid or reject public education messages aimed at reducing the use of tobacco products [28]. Thus, there is a need to understand how to increase young adults’ receptivity towards such anti-tobacco messaging. One way to start working towards this goal is to understand which social media sites are most often used by young adults, especially those who use e-cigarettes, and the extent to which young adults may be willing to engage with specific e-cigarette content. This understanding can then be integrated into new and existing public health campaigns and education interventions. The ultimate goals would be to increase digital health literacy among young populations, promote active engagement with social media and online content regarding health-related messages, and empower young adults to change to healthier behaviors [29]. In other words, digital health literacy programs could benefit from information regarding e-cigarette use status and social media use preference as well as information about engaging with e-cigarette health information on social media [6,21,29,30,31], to help prevent and reduce young adult e-cigarette use.

Because social media provide features, such as “Commenting”, “Liking”, and “Resharing”, to facilitate engagement with posts about e-cigarette health information [17,32], they are important avenues for research that is lacking in the literature. Active engagement with health information on social media can promote healthy behaviors, especially for young people [33]. Understanding engagement on social media may give us a more nuanced view of the association between social media use and e-cigarette perceptions among this young adult population. Additionally, because e-cigarette use is so prevalent in the young adult population, it is important to understand how use of the product is associated with young adults’ use of social media.

### 1.2. Study Purpose

The purpose of this exploratory study was to identify which behavioral and sociodemographic factors were associated with intentions to engage, in different methods (“Comment on”, “Re-share”, “DM (Direct Message)/Send this ad to a friend”, “Like”, and “Take a screenshot of”), with anti-vaping Instagram posts among a young adult population. Instagram is a free photo and video sharing application that is available on smart phones and online web browsers. People who use Instagram can post photos and videos and follow friends on the application, and are also exposed to content that the Instagram algorithm thinks may be interesting to specific audiences. People must be 13 years or older to sign up for the application. Instagram was chosen because it is a popular platform for e-cigarette social media posts and a platform that young adults frequently use [31]. We proposed the following research questions:

**Research Question 1**. *Does e-cigarette use status influence engagement intentions with Instagram posts?*

**Research Question 2**. *How are e-cigarette use and social media/internet use associated?*

Given the literature around the association between exposure to online content around substance use and substance use behaviors among young adults [8,14], we additionally hypothesized the following:

**Hypothesis 1**. *Past 30-day e-cigarette use will be associated with less intention to engage with anti-vaping social media posts.*

**Hypothesis 2**. *Past 30-day e-cigarette use will be associated with use of more social media sites and more internet use.*

Findings from this study can inform the design and targeting of future social media campaigns as well as health digital literacy initiatives to help prevent and reduce young adult e-cigarette use.

## 2. Materials and Methods

### 2.1. Participants

We recruited a convenience sample of young adults in July of 2022 into an online experimental study from Prolific (Prolific.co), an international crowdsourcing platform for behavioral research. Participants were eligible if they were between 18 and 30 years old and lived in the United States, and no other exclusion criteria were applied. We included young adults aged 18 to 20, despite the legal age of purchase being 21, due to the high levels of e-cigarette use in this age group [34]. We also intentionally recruited a balanced sample of participants who reported past 30-day e-cigarette use and nonuse, using the following two prescreening questions. We first asked participants “Have you ever used an electronic nicotine product, even one or two times? (Electronic nicotine products include e-cigarettes, vape pens, personal vaporizers and mods, pod-based, e-cigars, e-pipes, e-hookahs and hookah pens.” with the answer options of Yes/No. If participants selected “Yes”, they were then asked, “During the past 30 days, on how many days did you use e-cigarettes?” with a dropdown answer option from 0 to 30.

### 2.2. Procedure

Participants reviewed a brief description about the study on Prolific before being directed to the survey on Qualtrics. After providing consent, participants were asked to complete questions about their e-cigarette and other tobacco use, and social media/internet use. Then, participants saw five image-based Instagram posts about the health harms of using e-cigarettes. After viewing the posts, participants completed an attention check that validated what they viewed in the posts, by asking what the source of the Instagram image was (expert, friend, or influencer). Participants were then asked questions about their perceptions of the Instagram posts, perceived harms and intentions to use e-cigarettes, and their demographics. After completion, participants were compensated USD 3.50 via Prolific policies. All procedures were approved by UMass Chan Medical School’s Institutional Review Board.

### 2.3. Stimuli

For the five Instagram posts shown to participants, we used images from The Real Cost (https://therealcost.betobaccofree.hhs.gov/vapes/real-facts (accessed on 6 December 2021)), a public anti-vaping campaign aimed to educate youth and young adults about the harms of using e-cigarettes [27]. When showing our stimuli, we simulated the Instagram use experience by formatting the posts in the aesthetic style of Instagram’s platform and vertically presenting posts to scroll to view all five images. Posts included messages that said “Vapes can contain acrolein which can cause irreversible lung damage” and “Vapes can expose you to nicotine, which can cause cravings and other symptoms of addiction”. Images of the five Instagram posts were exactly the same for all participants, with the exception of the source. The source refers to the handle or account from which the post was posted. Participants were randomized to see the posts from one of three possible sources: expert, friend, or influencer. The examination of the effect of source is reported elsewhere.

### 2.4. Measures

#### 2.4.1. Engagement Intentions

Our main outcome variable was engagement intentions with the Instagram posts. Participants were asked if they would likely “Comment on”, “Reshare”, “DM (Direct Message)/Send this ad to a friend”, “Like”, and/or “Take a screenshot of” all five of the posts they viewed. An overall engagement intention score was created by summing the total of each type of engagement outcome (“Comment on”, “Re-share”, “DM (Direct Message)/Send this ad to a friend”, “Like”, and “Take a screenshot of”) they selected, with each type of engagement given equal weight for a possible range of 0 (none selected) to 5 (all selected) [17].

#### 2.4.2. Social Media Use

Participants were presented with a list of social media sites and asked “Which of the following social media platforms do you use? Please choose all that apply” with the answer options of YouTube, Instagram, Snapchat, Facebook, Twitter, Tumblr, Reddit, TikTok, WhatsApp, Pinterest, LinkedIn, Discord, other social media, and no social media [35]. A total social media sites used variable was created by summing up the number of platforms that participants indicated using from the presented list (range 0–13) [17].

#### 2.4.3. Daily Internet Use

Participants were asked about their daily internet use: “On average, how many hours do you spend on the internet each day (on a computer, mobile phone, or other device)? Please enter a number from 0–24” [17].

#### 2.4.4. E-Cigarette Use Status

Participants were asked to report their e-cigarette use in the two prescreening questions. The item asked if they had ever “used an electronic cigarette (e-cigarette), even one or two times?” If participants answered “Yes”, they were then asked, “During the past 30 days, on how many days did you use an e-cigarette?” [36]. E-cigarette use status was categorized as “past 30-day” use if they used an e-cigarette in the past 30 days, “ever” use if they ever used e-cigarettes but reported 0 days of e-cigarette use in the past 30-days, and “never” use if they responded “No” to ever using an e-cigarette even one or two times [36].

#### 2.4.5. Other Tobacco Use Status

Participants reported their use of combustible cigarettes, cigars, smokeless tobacco, and hookah. First, participants were asked about ever use of combustible cigarettes (“Have you ever smoked a tobacco cigarette, even just one puff?”), cigars (“Have you ever smoked cigars, cigarillos, or little cigars, even just one puff?), smokeless tobacco (“Have you ever used smokeless tobacco, even just once?”), and hookah (“Have you ever smoked hookah, even just one puff?”) Then, for each tobacco product, if participants indicated any ever use, they were asked how many days during the past 30 days they used the product, with a dropdown answer option from 0 to 30.

For each product, use status was categorized as “past 30-day use” if they used the product in the past 30 days, and “no past 30-day use” if they did not use the product in the past 30 days. We then combined all types of tobacco use (cigarettes, cigar, smokeless, and hookah) together as one combined “other tobacco use status” variable that represented any past 30-day use of the four tobacco products. Other tobacco use status was categorized as “past 30-day” use if they used any of the four tobacco products in the past 30-days, and “no past 30-day” use if they used none of the four products in the past 30-days. We kept other tobacco use status as a binary variable to prevent small cell sizes [36].

#### 2.4.6. Sociodemographic Characteristics

Participants reported their age with the question “How old are you?” and a dropdown with 18 to 30. Participants reported their gender identity, recoded to female, male, and nonbinary. Race/ethnicity were asked as a single item: “What race/ethnic group do you identify with? (check all that apply)” with the possible answer options of Asian-Eastern, Asian–Indian, Black/African American, Native American or Alaskan Native, Pacific Islander, White/Caucasian, Hispanic/Latino, and Other. We then recoded race/ethnicity to four categories (Non-Hispanic White/Caucasian, Non-Hispanic Black/African American, Other/Multiple, and Hispanic/Latino) to prevent small cell sizes.

### 2.5. Statistical Analysis

Analyses were conducted using R software [version 1.1.456] [37]. We ran descriptive statistics of the demographic variables, as well as use of each of the social media sites and each of the engagement intentions outcomes.

To model engagement intentions, we used logistic regression to run adjusted models for each engagement outcome that included fixed effects for age, race/ethnicity, gender, e-cigarette use status, other tobacco use status, source of Instagram post (friend, expert, influencer), total number of social media sites used, and daily internet use. For the sum engagement intentions outcome, we used Poisson regression to treat the outcome as a count variable [17]. We also ran adjusted logistic and Poisson models including product interaction terms (1) between e-cigarette use and total social media sites variables; and (2) between e-cigarette use and daily internet use variables. Statistical significance of fixed effects, including the interaction terms, was assessed using a partial F-test with an alpha of 0.05, and 95% confidence intervals (CI) were assessed. We adjusted for multiple comparisons using the Bonferroni correction [38].

To examine the association between e-cigarette use status and use of social media, we conducted chi-squared tests comparing e-cigarette use status with (1) use of each individual social media site, (2) total number of social media sites used, and (3) daily internet use. We then used logistic regression to run adjusted models for each social media site outcome that included fixed effects for e-cigarette use status, age, race/ethnicity, gender, and other tobacco use status. We used linear regression to run adjusted models for the continuous outcomes of total number of social media sites used and hours of daily internet use.

## 3. Results

### 3.1. Participants

Of the 510 participants who began our survey, our analytic sample included the N = 459 participants who completed the survey. The sample size was sufficient to meet adequate statistical power for the experimental study conducted as part of the survey. The average age of the sample was 24.6 years old (SD = 3.4), approximately half were male (47.1%), and the majority of participants identified as Non-Hispanic White/Caucasian (64.1%). Over half (54.9%) reported past 30-day e-cigarette use, and one-third (33.6%) reported past 30-day use of other tobacco products. See Table 1 for the complete demographics of the study participants.

### 3.2. Social Media/Internet Use

The top social media platforms that participants reported using were YouTube (n = 426, 92.8%), Instagram (n = 385, 83.9%), and Reddit (n = 335, 73.0%). Participants reported using an average of 6.6 (SD = 2.2) social media sites in total and spending an average of 7.1 h (SD = 3.8) on the internet every day.

### 3.3. Engagement Intentions

Participants indicated that they would “Comment on” the Instagram posts they saw (n = 51, 11.1%), “Reshare” (n = 37, 8.1%), and “DM/Send this ad to a friend” (n = 72, 15.9%). Over half of participants (n = 259, 56.4%) said they would “Like” the Instagram posts they saw, and a quarter of participants (n = 113, 24.6%) said they would “Take a screenshot” of the post. Of the five types of engagement, participants, on average, reported intentions to engage in 1.61 (SD = 0.69) types of ways.

### 3.4. Predictors of Engagement Intentions

Adjusted models that included product interaction terms between (1) e-cigarette use and total social media site variables, and (2) e-cigarette use and daily internet use were not significant; thus, all models were run without interaction terms (Table 2).

The odds of intention to “Comment on” the Instagram posts was higher among those with higher daily internet use (OR: 1.10, CI: 1.02–1.19, *p* = 0.016) and of older age (OR: 1.10, CI: 1.01–1.22, *p* = 0.040). The odds of intention to “Like” the Instagram posts was higher among those who used more social media sites (OR: 1.11, CI: 1.01–1.22, *p* = 0.025), and lower for those who identified as nonbinary (OR: 0.26, CI: 0.09–0.63, *p* = 0.006) (vs. female) and had higher daily internet use (OR: 0.92, CI: 0.89–0.99, *p* = 0.019). A higher sum engagement intention score was associated with higher total number of social media sites used (OR: 1.05, CI: 1.01–1.09, *p* = 0.019). Intentions to “Reshare”, “DM/Send this ad to a friend”, and “Take a screenshot of” the Instagram posts were not significantly associated with any sociodemographic characteristics.

### 3.5. E-cigarette Use Status and Social Media/Internet Use

E-cigarette use status differed significantly for Snapchat (X-squared = 6.3, df = 2, *p*-value = 0.043) and TikTok (X-squared = 16.1, df = 2, *p* < 0.001), as young adults who reported past 30-day e-cigarette use (Snapchat: n = 180, 70.3%; TikTok: n = 173, 72.3%) were more likely to use these two platforms compared to young adults who reported never use of e-cigarettes (Snapchat: n = 63, 42.0%; TikTok: n = 51, 34.0%) and ever use of e-cigarettes (Snapchat: n = 68, 66.0%; TikTok: n = 64, 62.1%). E-cigarette use status did not differ significantly for the other social media sites, as well as total number of social media sites used, and daily internet use.

Table 3 displays the results of the adjusted regression models predicting each type of social media site use. Young adults who reported past 30-day e-cigarette use had higher odds of using Twitter (OR: 2.03, CI: 1.16–3.56, *p* = 0.013) and TikTok (OR: 3.31, CI: 1.86–5.97, *p* < 0.001) compared to young adults who reported never use of e-cigarettes. Young adults who reported past 30-day e-cigarette use were more likely to use a higher number of social media sites (b: 0.58, CI: 0.01–1.14, *p* = 0.046), compared to young adults who reported never use of e-cigarettes. Young adults who reported ever use of e-cigarettes had higher odds of using TikTok (OR: 2.46, CI: 1.35–4.55, *p* = 0.004), compared to young adults who reported never use of e-cigarettes. Young adults who reported ever use of e-cigarettes were more likely to use the internet for a lower number of hours per day (b: −1.31, CI: −2.33–−0.28, *p* = 0.012), compared to young adults who reported never use of e-cigarettes.

## 4. Discussion

Overall, we found that specific types of intentions to engage with the anti-vaping Instagram posts were associated with social media use characteristics, and those who used more social media sites were more likely to report intentions to engage with the Instagram posts. This suggests that the quantity of time spent on social media may have differing effects on different types of engagement, yet exposure to more types of social media platforms encourages more intentions to engage with posts among young adults. Thus, anti-vaping campaigns may consider being on multiple social media platforms to have not only extended reach but also higher engagement with the anti-vaping content. Interestingly, daily internet use had mixed associations with different types of engagement intentions, such that higher internet use was associated with greater intentions to “Comment on”, but lower intentions to “Like” the posts. To “Comment on” a post is a more active form of social media engagement than simply clicking “Like”; therefore, public health officials may leverage online discussion forums that require more active engagement among audience members to reach young people who spend more time online with health education messages [39,40]. Contrary to our hypothesis, we did not find e-cigarette use status to be associated with any of the engagement outcomes. Thus, additional research is warranted in the field to better understand the ways to best engage with young adults, especially those who use e-cigarettes, when delivering anti-vaping messaging on popular social media sites.

We found that young adults who reported past 30-day e-cigarette use were more likely to have a higher number of total social media sites used, which was consistent with our hypothesis. Overall, we found that YouTube, Instagram, and Reddit were the top social media platforms used by our participants, consistent with prior research on popular social media sites among young adults [16]. Yet, e-cigarette use status was significantly associated with the use of two specific social media platforms: TikTok and Twitter. Young adults who reported past 30-day e-cigarette use were more likely to use these platforms compared to young adults who reported never use of e-cigarettes. These sites differed from the top two most popular platforms among the entire sample, suggesting that certain platforms, even if not the most widely used, attract young adults who have used e-cigarettes in the past 30-days. It shows that media channel choices might be important for targeting specific use status groups when addressing prevention and e-cigarette cessation.

However, contrary to our hypothesis, we found that ever e-cigarette use was associated with less time spent on the internet (vs. never e-cigarette use), and there were no significant associations with past 30-day e-cigarette use and time spent on the internet. This finding further points towards the need to understand how specific internet sites (including social media) are associated with e-cigarette use among young adults.

Our study has its limitations. Our stimuli only focused on one social media site, Instagram, because Instagram is one of the most popular platforms of e-cigarette social media marketing and a platform that young adults use [31]. Future studies should aim to understand factors associated with engagement with different types of popular social media sites, such as TikTok or Reddit, that contain different features and ways to engage with content. In asking participants of their likelihood to “Comment on” the posts, we did not assess what types of comments they would leave (e.g., negative, positive, or neutral). A prior study that examined the comments of social media tobacco prevention campaign messages have found that comments with a positive tone were associated with greater agreement with the campaign messages, in contrast to comments with a negative tone that were associated with the message disagreement [41]. Future research could examine the association of the tone of comments for the e-cigarette prevention social media posts to better understand its association with young adult engagement with e-cigarette social media messages.

Our study also has theoretical implications. We also used self-report measures to assess engagement intentions, rather than measuring actual engagement with the Instagram posts. However, the theory of planned behavior poses that intentions are predictors of behavior [42], and our measures of intentions to engage can be an indicator for actual engagement with the Instagram posts [17]. However, the results of our study can be strengthened by assessing the real-world behavioral outcomes. Future research that can mock social media sites and experimentally test engagement would further support these findings. Yet, these findings provide preliminary research that can inform future longitudinal studies with causal research questions that aim to identify longer-term effects between social media exposure and e-cigarette use among young adults. Although certain social media sites included in our study, such as TikTok and Twitter, tend to be popular among a younger age group [43], we adjusted for age in our analyses and this was unlikely to affect our results.

Finally, there is a potential bias with using an online convenience sample, as results are not representative and thus are not generalizable. However, use of an online panel of convenience sample is appropriate for conducting exploratory analysis [44], as supported by the past studies examining e-cigarette prevention messages with young adults [45]. As such, the preliminary results from our exploratory research provide evidence to inform the development of a large trial using a representative sample.

## 5. Conclusions

In conclusion, social media anti-vaping campaigns may be an effective method of engaging younger audiences, a generation that frequents social media [16,31]. Efforts to disseminate social media anti-vaping campaigns should launch on multiple platforms that are popular among young adults who use e-cigarettes, such as TikTok and Twitter, but especially TikTok since it was popular for both young adults who reported ever and past 30-day e-cigarette use. While social media has contributed to the popularity of e-cigarettes among young adults [2,13], it can also provide a method of proliferating anti-vaping messages to a captive audience. Considering that young adults avidly use social media [16,31], these findings support how existing anti-vaping campaigns may indeed be a useful tool for preventing the e-cigarette initiation and use among young people [46]. The initial evidence from exploratory research using a convenience sample can inform the development of a larger trial using a representative sample. Further research should continue to explore how to best engage people who use e-cigarettes with anti-vaping messages on social media.

## Figures and Tables

**Table 1:** Participant demographics (N = 459).

	n (%)
Age; M (SD)	24.6 (3.4)
Gender	
Female	216 (47.1)
Male	219 (47.7)
Nonbinary ^a^	24 (5.2)
Race/ethnicity	
Non-Hispanic White/Caucasian	294 (64.1)
Non-Hispanic Black/African American	24 (5.2)
Other ^b^/Multiple	97 (21.1)
Hispanic/Latino	44 (9.6)
Other tobacco use	
Past 30-day	154 (33.6)
E-cigarette Use	
Never	107 (23.3)
Ever	100 (21.8)
Past 30-day	252 (54.9)
Total number social media sites; ^c^ M (SD)	6.6 (2.2)
Total internet use; M (SD)	7.1 (3.8)

Notes: ^a^ Nonbinary includes trans female/trans woman, trans male/trans man, gender queer/gender nonconforming/gender expansive. ^b^ Others include Asian, Native American or Alaskan Native, or Pacific Islander. ^c^ Total number of social media sites include the following possible social media sites: YouTube, Instagram, Snapchat, Facebook, Twitter, Tumblr, Reddit, TikTok, WhatsApp, Pinterest, LinkedIn, Discord, Other.

**Table 2:** Adjusted regression results for each engagement outcome, OR (95% CI).

	“Comment on”	“Reshare”	“DM/Send This ad to a Friend”	“Like”	“Take a Screenshot of”	Sum Engagement Score
**E-cig use**						
Never (ref)						
Ever	1.35 (0.43–4.43), 0.612	1.16 (0.42–3.29), 0.769	0.65 (0.29–1.42), 0.280	0.92 (0.51–1.69), 0.798	0.72 (0.35–1.44), 0.350	0.93 (0.71–1.20), 0.563
Past 30-day	2.67 (1.00–8.05), 0.062	0.69 (0.24–1.98), 0.479	0.71 (0.34–1.46), 0.344	0.58 (0.33– 1.01), 0.055	1.24 (0.67–2.30), 0.497	0.94 (0.73–1.19), 0.591
**Age**	1.10 (1.01–1.22), 0.040	1.02 (0.91–1.13), 0.766	0.98 (0.91–1.06), 0.617	0.97 (0.92–1.03), 0.380	1.02 (0.96–1.09), 0.481	1.01 (0.98–1.03), 0.686
**Gender**						
Female (ref)						
Male	2.08 (1.06–4.22), 0.036	1.23 (0.60–2.57), 0.573	1.26 (0.74–2.17), 0.395	0.69 (0.46–1.04), 0.380	1.02 (0.64–1.63), 0.934	1.02 (0.85–1.23), 0.814
Nonbinary	1.05 (0.15–4.43), 0.950	NA	0.46 (0.07–1.72), 0.314	0.26 (0.09–0.63), 0.006	1.98 (0.77–4.88), 0.143	0.74 (0.45–1.14), 0.191
**Race**						
Non-Hispanic White/Caucasian (ref)						
Non-Hispanic Black/African American	1.11 (0.29–3.46), 0.870	3.85 (1.15–11.21), 0.018	1.47 (0.46–3.95), 0.471	1.72 (0.70–4.56), 0.250	1.16 (0.40–2.94), 0.765	1.32 (0.92–1.84), 0.117
Other/Multiple	0.72 (0.29–1.60), 0.444	1.34 (0.52–3.18), 0.520	1.44 (0.78–2.59), 0.227	0.95 (0.58–1.56), 0.847	1.52 (0.89–2.56), 0.120	1.10 (0.89–1.36), 0.382
Hispanic/Latino	0.62 (0.17–1.79), 0.418	2.41 (0.82–6.31), 0.086	0.38 (0.09–1.12), 0.122	1.14 (0.59–2.27), 0.697	1.18 (0.54–2.43), 0.669	1.01 (0.74–1.35), 0.948
**Other tobacco use**						
Never (ref)						
Past 30-day	1.41 (0.70–2.89), 0.343	1.40 (0.58–3.42), 0.453	1.28 (0.67–2.44), 0.429	0.75 (0.47–1.21), 0.246	1.01 (0.59–1.72), 0.981	1.03 (0.83–1.28), 0.776
**Source**						
Expert (ref)						
Friend	1.87 (0.85–4.24), 0.122	1.40 (0.58–3.47), 0.458	0.99 (0.53–1.85), 0.974	0.95 (0.59–1.53), 0.845	0.91 (0.53–1.55), 0.721	1.05 (0.85–1.29), 0.681
Influencer	1.78 (0.82–4.00), 0.150	1.48 (0.63–3.62), 0.372	0.94 (0.50–1.75), 0.839	1.21 (0.75–1.94), 0.439	1.00 (0.59–1.70), 0.987	1.11 (0.90–1.36), 0.346
**Total number** **social media sites**	0.92 (0.80–1.06), 0.261	1.14 (0.97–1.36), 0.110	1.13 (1.00–1.28), 0.053	1.11 (1.01–1.22), 0.025	1.09 (0.98–1.21), 0.101	1.05 (1.01–1.09), 0.019
**Daily internet use**	1.10 (1.02–1.19), 0.016	0.99 (0.89–1.08), 0.791	1.00 (0.93–1.07), 1.000	0.92 (0.89–0.99), 0.019	0.98 (0.92–1.04), 0.579	0.99 (0.97–1.02), 0.526

Note. Odds ratios, 95% confidence intervals, and *p*-values are presented.

**Table 3:** Adjusted regression results of e-cigarette use status predicting social media site use.

	Ever Use (OR, 95% CI)	Past 30-Day Use (OR, 95% CI)
YouTube	2.13 (0.62–8.48), 0.244	1.02 (0.37–2.71), 0.963
Instagram	1.02 (0.48–2.14), 0.966	1.10 (0.54–2.23), 0.789
Snapchat	1.63 (0.88–3.02), 0.121	1.81 (1.01–3.26), 0.045
Facebook	1.03 (0.56–1.89), 0.925	1.08 (0.61–1.90), 0.803
Twitter	1.23 (0.69–2.20), 0.481	2.03 (1.16–3.56), 0.013
Tumblr	1.76 (0.75–4.28), 0.200	1.07 (0.47–2.52), 0.870
Reddit	1.22 (0.63–2.35), 0.557	1.23 (0.67–2.23), 0.498
TikTok	2.46 (1.35–4.55), 0.004	3.31 (1.86–5.97), <0.001
WhatsApp	1.28 (0.58–2.82), 0.541	1.58 (0.78–3.33), 0.216
Pinterest	1.95 (0.99–3.89), 0.055	1.26 (0.67–2.38), 0.467
LinkedIn	1.02 (0.57–1.84), 0.942	0.83 (0.47–1.45), 0.506
Discord	0.72 (0.39–1.32), 0.288	0.68 (0.39–1.20), 0.188
Total Number Social Media Sites Used (b, 95% CI)	0.54 (−0.06–1.14), 0.080	0.58 (0.01–1.14), 0.046
Total Number of Hours Online Every Day (b, 95% CI)	−1.31 (−2.33, −0.28), 0.012	−0.17 (−1.47, 0.44), 0.293

Note. Odds ratios presented for individual social media sites, estimates presented for total number of social media sites and daily internet use; 95% confidence intervals presented with *p*-values. Models were additionally adjusted for age, gender, race/ethnicity, and other tobacco use. The refence group for e-cigarette use status was never use. Total number of social media sites used and total number of hours online every day were operationalized as continuous outcomes; thus, risk ratios are presented.

## Data Availability

The data presented in this study are available on request from the corresponding author.
